# Microplastic-Free Microcapsules Using Supramolecular Self-Assembly of Bis-Urea Molecules at an Emulsion Interface

**DOI:** 10.1021/acs.langmuir.4c00541

**Published:** 2024-07-11

**Authors:** Siddhant Pravin Bhutkar, Pierre-Eric Millard, Jon A. Preece, Zhibing Zhang

**Affiliations:** † School of Chemical Engineering, University of Birmingham, Birmingham B15 2TT, U.K.; ‡ BASF SE, Ludwigshafen Am Rhein 67056, Germany; § School of Chemistry, University of Birmingham, Birmingham B15 2TT, U.K.

## Abstract

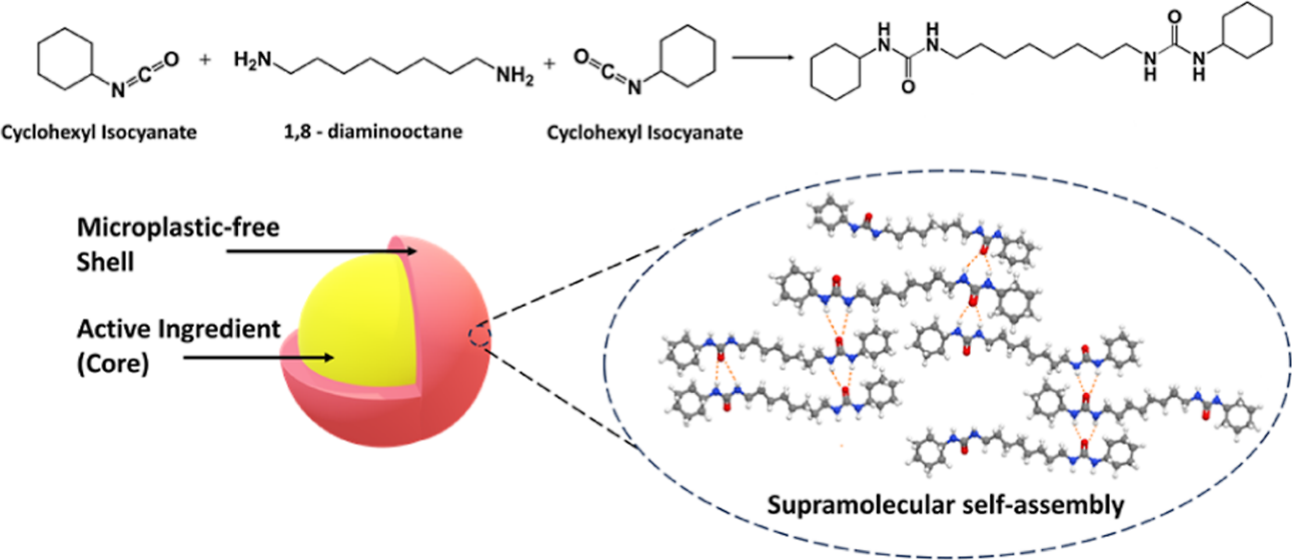

Encapsulation technology is well established for entrapping active ingredients within an outer shell for their protection and controlled release. However, many solutions employed industrially use nondegradable cross-linked synthetic polymers for shell formation. To curb rising microplastic pollution, regulatory policies are forcing industries to substitute the use of such intentionally added microplastics with environmentally friendly alternatives. This work demonstrates a one-pot process to make microplastic-free microcapsules using supramolecular self-assembly of bis-ureas. Molecular bis-urea species generated *in-situ* spontaneously self-assemble at the interface of an oil-in-water emulsion via hydrogen bonding to form a shell held together by noncovalent bonds. In addition, Laponite nanodiscs were introduced in the formulation to restrict aggregation observed during the self-assembly and to reduce the porosity of the shell, leading to well-dispersed microcapsules (mean Sauter diameter *d* [3,2] ∼ 5 μm) with high encapsulation efficiency (∼99%). Accelerated release tests revealed an increase in characteristic release time of the active by more than an order of magnitude after encapsulation. The mechanical strength parameters of these capsules were comparable to some of the commercial, nondegradable melamine–formaldehyde microcapsules. With mild operating conditions in an aqueous environment, this technology has real potential to offer an industrially viable method for producing microplastic-free microcapsules.

## Introduction

Microencapsulation refers to trapping active ingredients (AIs), with a specific function in formulations, in a micrometer-sized enveloping shell. This technology finds widespread use in products like agrochemicals,^1^ perfumes and fragrances,^2^ food and flavors,^3^ detergents,^4^ cosmetics,^5^ and pharmaceuticals^6^ to name a few. These microcapsules help to protect the encapsulated AI from the external environment and ensure its controlled release in a timely manner. However, the shells of these microcapsules have often been designed using synthetic polymers that are nonbiodegradable and are therefore contributing to the global microplastic pollution challenge.^7,8^ The concern for microplastic pollution is rising among the public and governments.^9,10^ Along with their impact on the natural environment, the presence of microplastics in the human body (via food ingestion and air inhalation) is alarming.^11,12^ This global concern has led to new legislations worldwide compelling the use of environmentally friendly alternatives along with effective management and reduction of plastic waste.^13^ Specifically, since September 27, 2023, the European Chemicals Agency (ECHA) has imposed extensive Europe wide restriction which bans the use of intentionally added microplastics in products.^14^ This ban directly affects the use of microcapsules, especially in agricultural, cosmetic, and detergent formulations. Therefore, developing microplastic-free microcapsules is crucial for allowing sustainable industrial development.

Natural polymers such as starch, cellulose derivatives, gum Arabic, alginate, and proteins like gelatin^15−17^ have commonly been used for encapsulating AI in food formulations. In principle, microcapsules prepared using these materials can be considered microplastic-free since they are not synthetic. Specifically, in food encapsulation, the release of AI in the mouth is quick and governed primarily by capsule rupture due to prevalent frictional forces;^18^ whereas, in products like agrochemicals and laundry detergents, microcapsules need to withstand harsher conditions for longer times and provide controlled release.^19^ In these situations, cross-linked synthetic nonbiodegradable polymers, e.g., aminoplasts like urea–formaldehyde (UF) and melamine–formaldehyde (MF) are widely used industrially.^20^

Synthetic polyesters represent another major class of biodegradable polymers employed for microencapsulation.^21^ These include polylactides, polyglycolide, poly(ε-caprolactone), and poly(lactide-*co*-glycolide). The ester linkages in these polymers can degrade enzymatically or by hydrolysis to yield nontoxic products.^22^ Solvent evaporation is the most common technique used for the preparation of these types of capsules. However, large volumes of halogenated solvents (e.g., dichloromethane) are commonly used in this method and need to be (i) used in controlled environmental conditions to limit human exposure to their vapors, (ii) recovered, and (iii) effectively reused/disposed, which increases the complexity and cost of operation of the process. Furthermore, the phase separation and evaporation of the organic solvent affect the porosity of the shell which can lead to a fast leakage of the AI.^19,23^

Interfacial polymerization can overcome these drawbacks and provide an organic solvent-free approach for preparing microcapsules. The method involves forming a stable emulsion of two immiscible phases followed by polymerization of complementary monomers at the droplet interface forming a polymeric membrane.^24^ Perignon et al. have outlined this membrane formation mechanism for polyamide-, polyurea-, polyurethane-, and polyester-based polymers, which are most commonly used as shell materials.^25^ Furthermore, studies highlighting the kinetics of the interfacial polymerization process, specifically for polyurea microcapsules, can be found in the literature.^26,27^ All in all, the interfacial polymerization technology is well established and leads to formation of microcapsules with high encapsulation efficiencies (EEs) and tunable release properties.^28^ However, despite their superior encapsulation and AI release performance, the lack of biodegradability of these microcapsules is a major issue.^19^

Another route for preparing microplastic-free capsules is the use of nonpolymeric inorganic colloids (e.g., SiO_2_) for constructing the microcapsule shell. Pickering emulsions were used to build such capsules, first by Velev et al. and then by Dinsmore et al. which paved the way for subsequent studies.^29,30^ The self-assembled colloidal nanoparticles at the emulsion interface can be fixed to form a robust shell by thermal sintering,^31^ polyelectrolyte adsorption,^32^ aqueous gelation of the droplets,^33^ subsequent polymerization,^34−36^ covalent cross-linking,^37^ or growing a metallic or inorganic shell externally.^38,39^ Although there is a wide variety of colloids available, long-term encapsulation of small molecules in many such colloidal capsules is challenging with retention of AI limited to only a few hours.^37,40^ Microcapsules with a metallic shell can provide a good barrier property and retention of AI,^38^ but they are expensive. Furthermore, building a stable Pickering emulsion usually involves modifying the emulsifier surface to optimize their affinity toward the dispersed phase which can be difficult.^41^

The research discussed hitherto represents some of the alternatives which can potentially replace conventional, polymeric nonbiodegradable microcapsules. Each of these has been investigated previously in the literature with their corresponding merits and demerits. In this study, we introduce the use of bis-urea molecules to synthesize microcapsules. We have designed a one-pot, *in-situ* process to generate these bis-urea moieties which spontaneously self-assemble at the interface of an oil-in-water emulsion. The resultant core–shell microcapsules can effectively encapsulate AI in the dispersed phase. Specifically, a monoisocyanate and a bis-amine were used to create bis-urea molecules with two urea-linkages as the hydrogen bonding motifs. These molecules self-assembled at the oil–water interface of an emulsion and encapsulated a cosmetic oil, hexyl salicylate (HS). The shell material is formed completely by virtue of supramolecular hydrogen bonds, without the use of any polymer, making the capsules microplastic free. To the best of our knowledge, such a technique of synthesizing microcapsules using small bis-urea molecules has not been reported so far. A recent study by Wilson-Whitford et al. demonstrates the use of molecules with urethane linkages to synthesize crystalline microcapsules.^42^ However, the technique utilizes a two-step process along with the use of an organic solvent, wherein shell formation takes place by virtue of solvent evaporation. The technology developed in this work uses a contrasting approach, wherein the process is one-pot and completely aqueous, making it greener and more sustainable. Bis-urea molecules are known to self-assemble into complex forms.^43,44^ We have used this ability of such molecules for encapsulation for the first time. By variation of the reagents used in the reaction, the structure of the resultant bis-urea molecule was controlled, which consequently affected the self-assembly at the oil–water interface and microcapsule formation. Furthermore, critical characteristics of the microcapsules, like morphology, size, mechanical strength, and release rates of the encapsulated HS have been determined. With a simple approach under benign conditions, this new process represents a sustainable approach to synthesize microplastic-free microcapsules which can replace the conventional nonbiodegradable capsules used in industrial formulations.

## Experimental Section

### Materials

Poly(vinyl alcohol) (PVA, *M*_w_ 13,000–23,000, 87–89% hydrolyzed), cyclohexyl isocyanate, 1,6-diaminohexane, 1,8-diaminooctane, melamine, HS, tris(2-aminoethyl)amine, 1-propanol, ethanol, and Nile red were all of analytical reagent grade, procured from Merck (Dorset, UK). LR white resin was supplied by Agar Scientific (Stansted, UK). Laponite nanodiscs (Laponite-RD) were purchased from BYK-Chemie (Wesel, Germany). Dibutyl adipate (commercial name: Cetiol B) and isopropanol (IPA) were procured from BASF SE (Ludwigshafen, Germany). All chemicals were used as received without further purification. Double distilled water was used for all of the experiments.

### Capsule Synthesis

For a typical batch, PVA (0.4 g) was dissolved in water (80 mL) to form the continuous phase in a beaker (250 mL, glass). HS (11.33 g) was mixed with cyclohexyl isocyanate (2 g) and Nile red dye (∼2 mg) to form the oil phase. The beaker with the continuous phase was placed in an ice bath (to avoid overheating), and the oil was emulsified into the continuous phase using a Silverson L4RT homogenizer equipped with an emulsor screen. The speed of rotation was 5000 rpm, and the rotation time was 2 min. The prepared emulsion was transferred to a double-glazed jacketed reactor equipped with 4 stainless steel baffles, maintained at 12 °C using a circulating water bath (model F33-HL, Julabo GmbH, Germany, EU). An overhead stirrer equipped with a six-blade Rushton turbine was used for stirring. A solution of di/tri-amine (isocyanate/amine molar ratio, 2:1/3:1 based on the amine) in water (10 g) was added dropwise to the emulsion using a syringe pump (Harvard Apparatus, Pump 11 Elite) over ∼15 min (flow rate ∼0.7 mL min^–1^). The temperature was maintained at 12 °C during the addition and then raised to 20 °C (at 1 °C/min). The stirring speed was kept constant at 200 rpm throughout. The reaction mixture was held at these conditions overnight (18 h). The resultant microcapsule slurry was stored and used for characterization.

For preparing capsules using the Laponite nanodiscs, first, in a beaker (250 mL, glass), nanodiscs (0.64 g) were dispersed in water (74 g) by using a Rushton turbine (1000 rpm), yielding a 0.8% (w/w) dispersion. Once homogeneous, the dispersion was further stirred at 2000 rpm using the Silverson homogenizer to break any remaining aggregates for 15 min. A solution of NaCl (47 mg) in water (2 g) was added to this dispersion, followed by a 10% (w/w) aqueous PVA solution (4 g). This dispersion was used as the continuous phase. The oil phase containing HS, cyclohexyl isocyanate, and Nile red dye was emulsified in this continuous phase using the Silverson L4RT at 8000 rpm for 5 min. Using this emulsion, the encapsulation procedure was repeated exactly as described above.

### Bright-Field and Fluorescent Microscopy

Bright-field microscopy was used for recording optical images by using a Leica DM500 microscope. Furthermore, for capturing images under UV light, the microscope was mounted with a Cool-LEDpE-300 series illumination source. Images were captured at regular time intervals to monitor the reaction. When the Nile red dye was included in the oil phase, images were recorded under UV light to reveal the fluorescence and the presence of oil. A H3 filter cube (BP420-490), a dichromatic mirror (510), and a suppression filter (LP 515) were applied. The blue excitation light maximum was around 460 nm.

### Scanning Electron Microscopy

The microcapsule slurry was diluted (1:20 w/w) with water and deposited onto a glass slide using a Polos 150i spin coater operating at 2000 rpm for 20 s. The glass slide with the capsules was then mounted onto an aluminum stub and sputter coated with gold (∼6 nm) using a Polaron Sputter Coater SC7640 with argon as the inert gas. For imaging, the stub was mounted onto a Hitachi TM 3030 Plus table-top scanning electron microscope and the images were recorded at a voltage of 15 kV.

### Transmission Electron Microscopy

Transmission electron microscopy (TEM) was conducted using a JEM-1400 (JEOL Ltd., Japan) electron microscope at 120 kV. For sample preparation, the microcapsule slurry (∼2 mL) was centrifuged, and the supernatant clear aqueous layer was decanted off and replaced with 70% (v/v) aqueous ethanol and mixed. Then, the centrifugation cycle was repeated, and the supernatant ethanol solution was replaced with 90% (v/v) aqueous ethanol. Subsequent mixing and centrifugation cycle was repeated with 100% ethanol to remove all water from the sample. The ethanol was then replaced by a 50:50 (v/v) ethanol: LR white resin solution. Finally, the solution was replaced with a 100% LR white resin and the resulting sample was left to cure overnight at 60 °C in the oven for embedding the capsules. Thin slices (∼90 to 150 nm) were sliced from the set resin using a Microtome (Reichert-Jung, Germany). One slice was placed onto copper grids (2 × 1 mm slot) and loaded into the TEM stage for imaging.^45^

### Particle Size Analysis

The size of the microcapsules was measured using a Malvern Mastersizer 2000 (Malvern Instruments Ltd., UK) based on laser diffraction. The data were treated according to the Mie theory by Mastersizer 2000 software using a universal model provided by the instrument supplier. A few microliters of the prepared microcapsule slurry were added into the dispersing unit of the instrument containing distilled water (100–150 mL) and stirred continuously at 1500 rpm.

### Release Studies

Accelerated release experiments were performed, using 36% (v/v) aqueous 1-propanol as the release medium, to measure the leakage of the core oil and determine barrier properties of the shell according to the method described by Baiocco et al.^46^ Briefly, ∼7–8 mg (which contained roughly 0.8 to 1 mg of HS) of the microcapsule slurry was placed inside a dialysis tube (length ∼5 cm, internal diameter ∼1 cm, molecular weight cutoff, 14 kDa). The tube was filled with 2 mL of the release medium and capped securely using pegs on both ends. Prior to use, the tubing was washed thoroughly at 60 °C and rinsed at ambient conditions with deionized water to remove the glycerol coating. This tube containing the capsules was dropped into a 250 mL Duran flask with a screw-cap lid containing the release medium (100 mL) and stirred continuously on a stirring plate with a magnetic bar. Aliquots (5 mL) were withdrawn at specific time intervals and replaced with fresh release medium (5 mL) to maintain a constant volume. The withdrawn aliquots were tested in a calibrated UV–visible spectrophotometer (CE 2021, Cecil Instruments) at 306 nm [lambda maximum (λ_max_) for HS] to measure the concentration of the core oil released. As a control experiment, pure unencapsulated HS (0.75 mg) in the release medium (2 mL) was placed inside the dialysis tube and the experiment was repeated.

### Encapsulation Efficiency and Payload

Microcapsule slurry (2 g) obtained after reaction was diluted with water (8 g) and filtered using a standard Buchner funnel and vacuum pump with a filter paper (Whatman, ∼2 μm pore size). The filtrate (water + unencapsulated HS) was collected and weighed carefully in a centrifuge tube. Dibutyl adipate (10 g) was mixed with this filtrate and mixed on a vortex mixer (IKA Genius 3) for 5 min to facilitate extraction of HS. Then, the mixture was centrifuged (Hettich Universal 320 R centrifuge) at 5000 rpm (2370 g force) for 2 min to separate and recover the dibutyl adipate (supernatant). This mixing and centrifugation cycle was repeated once more with fresh dibutyl adipate (10 g). Finally, the amount of HS extracted into dibutyl adipate was measured (*W*_1_) using a calibrated UV–visible spectrophotometer (Shimadzu UV-1800) at 306 nm (λ_max_ for HS).

To calculate the total amount of HS in the final microcapsule slurry, the microcapsule slurry (1 g) was added to pure IPA (100 mL) in a 150 mL glass flask with a screw cap. The flask was then placed in an ultrasonic water bath for 2 h to extract the encapsulated HS into IPA. After sonication, the IPA was filtered using a 0.2 μm syringe filter to separate the broken capsules/shell material. The amount of HS extracted from the slurry (1 g, HS_extract_) was measured using a calibrated UV–visible spectrophotometer (Shimadzu UV-1800). The total amount of HS (*W*_total_) present in 2 g slurry was thus calculated as
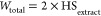
1and subsequently, the encapsulation efficiency (EE) was calculated as
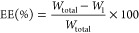
2where *W*_1_ represents the unencapsulated HS recovered from the filtrate water using dibutyl adipate.

The capsules collected after filtration were dried at room temperature overnight under the fume hood. Dry capsules (100 mg) were added to pure IPA (100 mL) in a glass flask (150 mL) with a screw cap. The flask was placed in an ultrasonic water bath (VWR Ultrasonicator, USC100TH) for 2 h to extract the encapsulated HS into IPA. After sonication, the dispersion was filtered using a 0.2 μm syringe filter to separate the broken capsules/shell material. The absorbance of the clear propanol solution was measured by using a calibrated UV–visible spectrophotometer (Shimadzu UV-1800) at 306 nm. The payload was calculated as follows
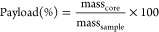
3where mass_core_ represents the mass of HS extracted into IPA and mass_sample_ represents the mass of dry capsules used for extraction. All of the experiments were performed in duplicate.

### Mechanical Strength

**Figure 1:**
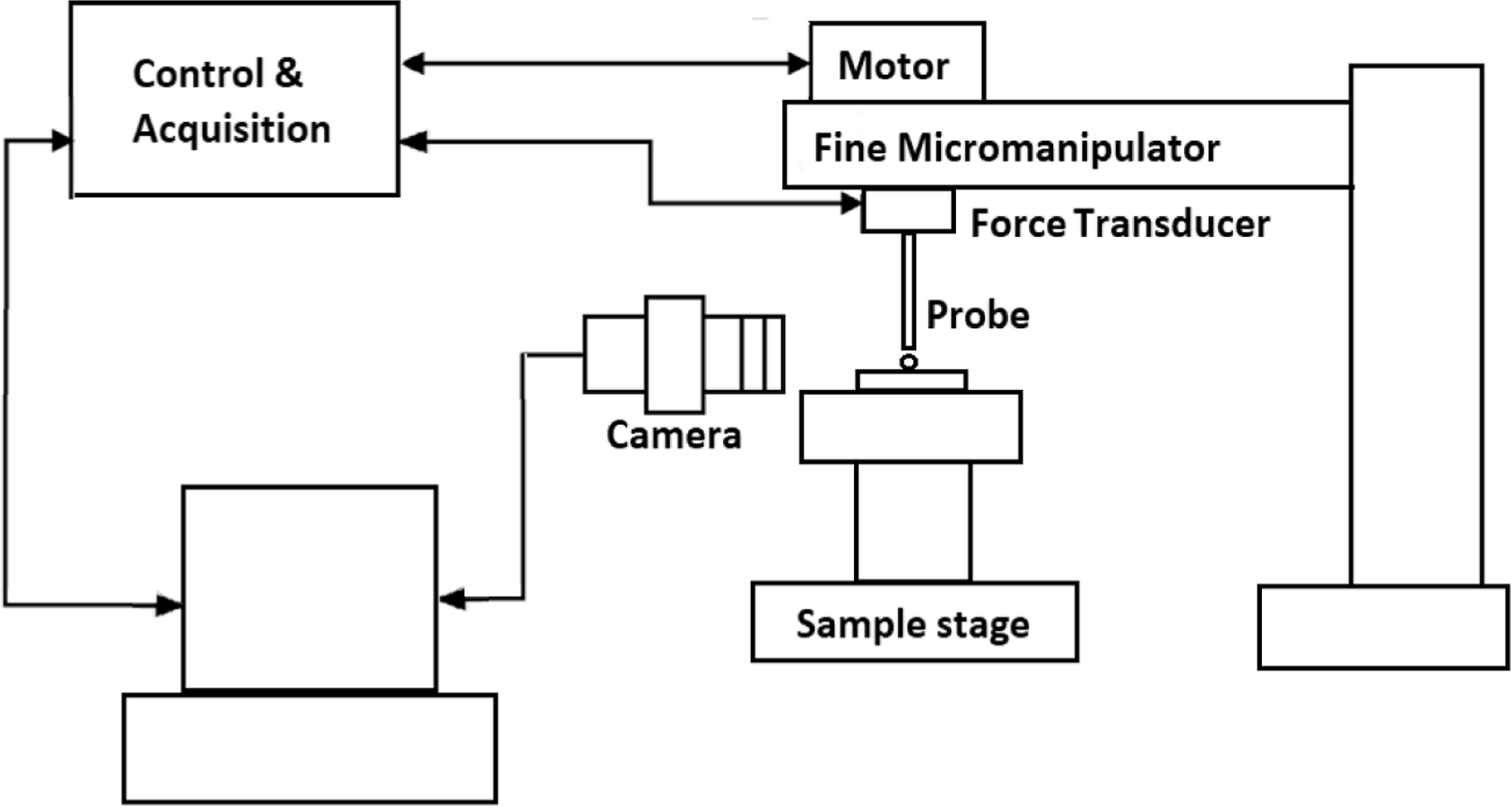
Schematic representation of the micromanipulation rig.

A micromanipulation technique (Figure 1) first developed in 1991 by Zhang et al. was used to test the mechanical strength of microcapsules.^47^ Originally used for measuring the rupture force of single mammalian cells, the technique has subsequently been modified and used for measuring the mechanical strength of single microcapsules.^48,49^ Briefly, a glass probe with a flat tip of ca. 70 μm (model 403A, Aurora Scientific Inc., Canada) was mounted onto a force transducer. The vertical movement of the probe was controlled by using a servo motor and a fine micromanipulator. The microcapsule slurry was sufficiently diluted (∼1:1000 w/w) with water and dried on a glass plate (ca. 2 cm^2^). The dilution ensured that individual capsules could be isolated for testing their mechanical strength. The glass slide was secured to the sample stage perpendicular to the probe. Thirty individual capsules chosen randomly were ruptured using the probe at a compression speed of 2 μm s^–1^. The voltage output recorded by the force transducer was converted into force using sensitivity (0.4939 mN V^–1^) which was determined beforehand by calibration.

## Results and Discussion

### Capsule Synthesis

**Figure 2:**
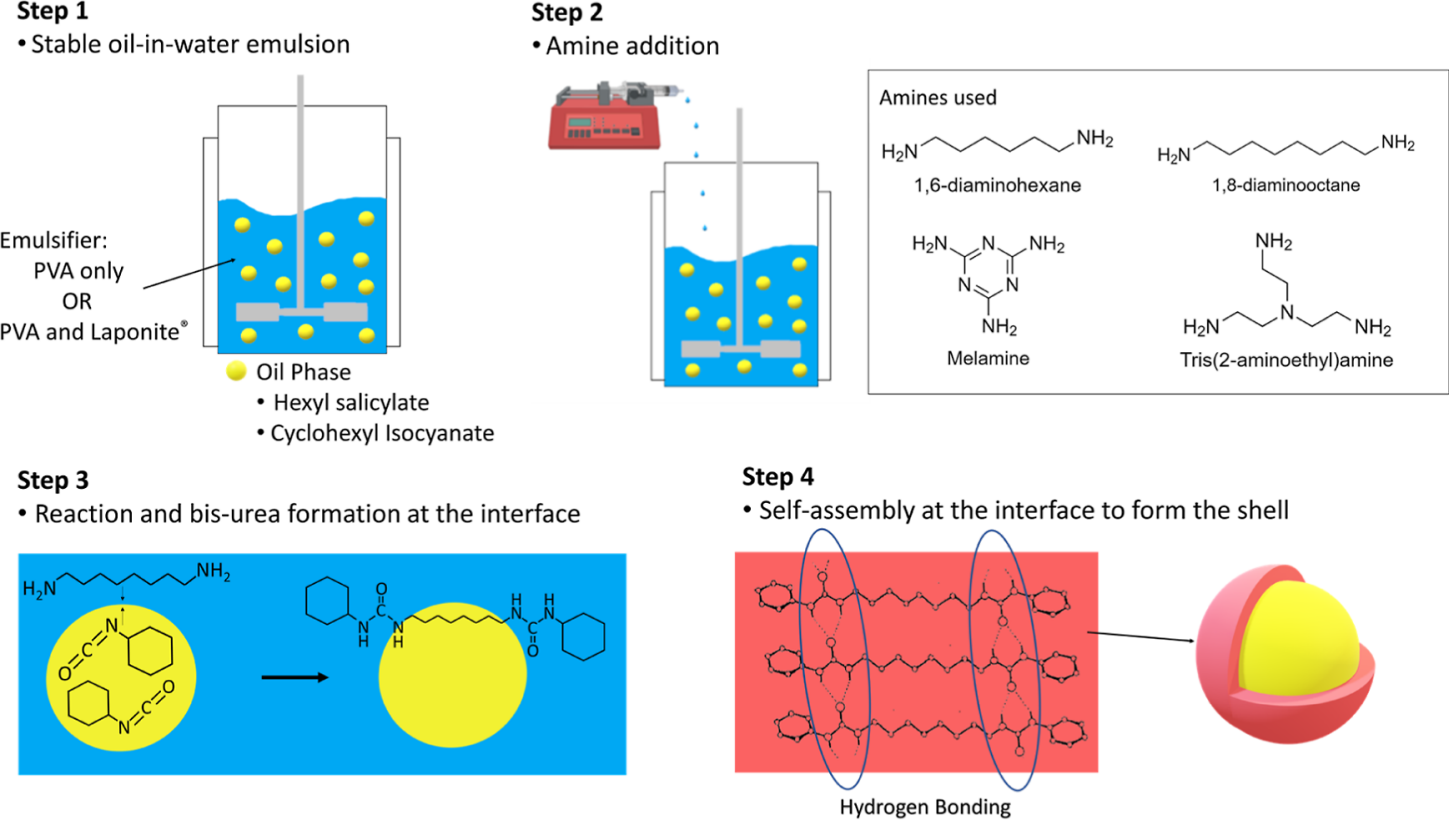
Schematic representation of the encapsulation process.

During the experiments, four different amines, 1,6-diaminohexane, 1,8-diaminooctane, melamine, and tris(2-aminoethyl)amine, were tested along with cyclohexyl isocyanate for shell formation. Figure 2 shows a schematic representation of the encapsulation process using cyclohexyl isocyanate and 1,8-diaminooctane as representative precursors. The oil phase containing HS and cyclohexyl isocyanate is emulsified in water (**step 1**), and the amine solution is added dropwise to the emulsion with stirring (**step 2**). The amine and isocyanate diffuse to the emulsion interface from the aqueous and oil phase, respectively, to form the molecular urea species (**step 3**), which in turn self-assemble to form the shell material across the surface of the HS oil droplets (**step 4**). Here, the self-assembly is solely driven by supramolecular hydrogen bonding, wherein, the urea-linkages act as the hydrogen-bonding motifs. Furthermore, by restricting the functionality of the isocyanate and amines, we ensure that long-chain polyurea formation does not occur.

**Figure 3:**
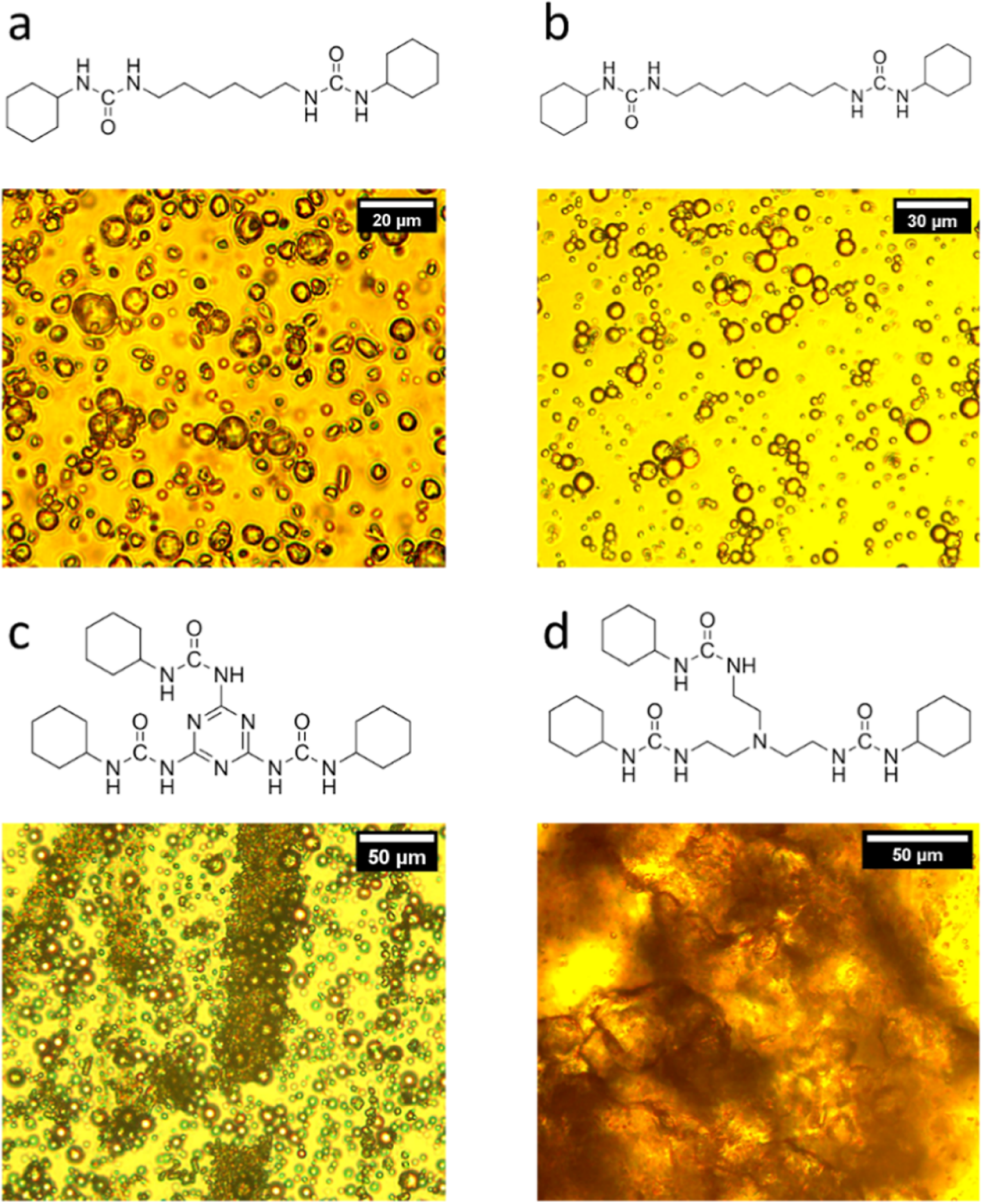
Microencapsulation attempts using cyclohexyl isocyanate and (a) 1,6-diaminohexane, (b) 1,8-diaminooctane, (c) melamine, and (d) tris(2-aminoethyl)amine.

Preliminary encapsulation attempts using the cyclohexyl isocyanate with each of the four amines were conducted using PVA as an emulsifier for stabilizing the emulsion. Figure 3 shows the proposed bis/tris molecular urea structures and the corresponding optical image of the reaction mixture for each system. The bifunctional ureas (Figure 3a,b) lead to the formation of microcapsules with shell formation at the oil–water interface. When melamine is used, the shell material phase separates from the emulsion and completely precipitates in the bulk leaving behind only HS in the dispersed droplets. In Figure 3c, this precipitated shell material can be seen as dark bands along with the emulsion droplets. Similarly, the shell material formed using tris(2-aminoethyl)amine is not confined to the interface and precipitates out, forming large aggregates (Figure 3d). Here, the trifunctional structures have shorter carbon chains and are bulkier (as compared to the bis-ureas) which presumably led to shell formation not confined to the interface. Therefore, the size and hydrophobicity of the molecule formed by the reaction between the isocyanate and the multifunctional amine are critical for the resultant interfacial assembly and shell formation.

**Figure 4:**
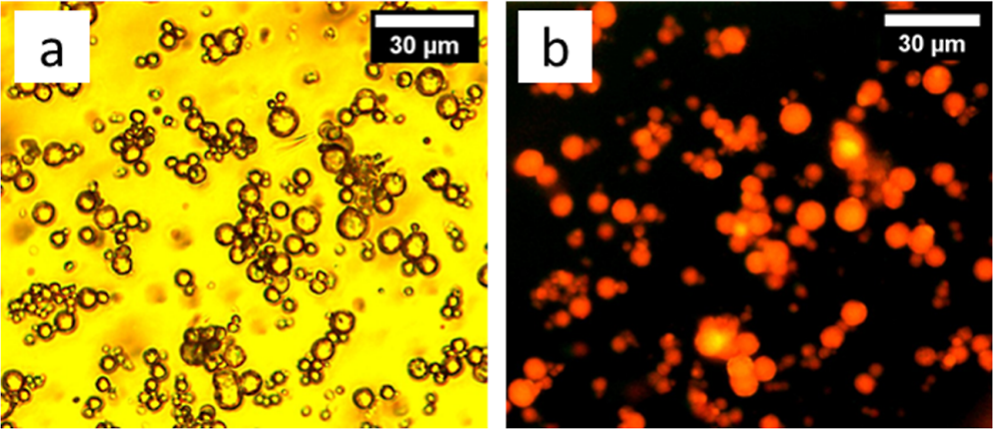
Images of microcapsules prepared using cyclohexyl isocyanate and 1,8-diaminooctane using (a) bright-field microscopy and (b) same image under UV light.

When the microcapsules prepared using 1,8-diaminooctane are compared under bright field (Figure 4a) and UV light (Figure 4b), the oil encapsulation is clearly visible, as evidenced by the intense Nile red dye emission (Figure 4b) incorporated into the lipophilic HS. The capsules prepared using 1,6-diaminohexane showed similar results.

**Figure 5:**
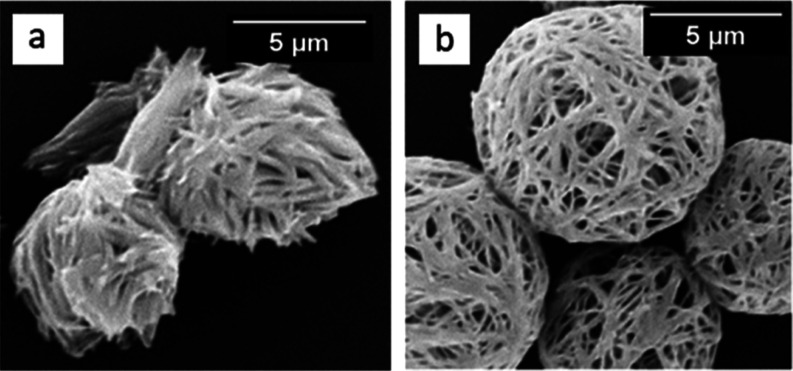
SEM images of microcapsules prepared using cyclohexyl isocyanate and using (a) 1,6-diaminohexane or (b) 1,8-diaminooctane as the diamine.

However, there was a distinct contrast between the SEM images of 1,6-diaminohexane (Figure 5a) and 1,8-diaminooctane (Figure 5b) bis-urea microcapsules. 1,6-diaminohexane capsules are nonspheroidal and needle-like aggregates are formed (Figure 5a); in contrast, microcapsules prepared using 1,8-diaminooctane show spheroidal interconnected continuous fiber-like structures which conform well to the spherical interface of the oil droplet (Figure 5b). Presumably, the extra ethylene moiety (1,6- vs 1,8-diamine) imparts greater flexibility during self-assembly along with improved hydrophobicity, forming a shell that can envelope the template oil droplet more readily in the case of 1,8-diaminooctane. This result implied that longer alkyl chains might provide even better shell-forming properties. Consequently, experiments were conducted with 1,10-diaminodecane and 1,12-diaminododecane (see Section S2 for details). However, these diamines themselves were sparingly soluble in water at room temperature. Nevertheless, during the experiment, they were added as solids to the emulsion (step 2, Figure 2) to check if simultaneous dissolution and reaction with the isocyanate in the dispersed phase can lead to microcapsule formation. In both cases, even if some microcapsules were formed, large lumps of presumably a mixture of bis-urea molecules and unreacted diamines were observed under the microscope (Figures S1 and S2).

### Particle Size Distribution

**Figure 6:**
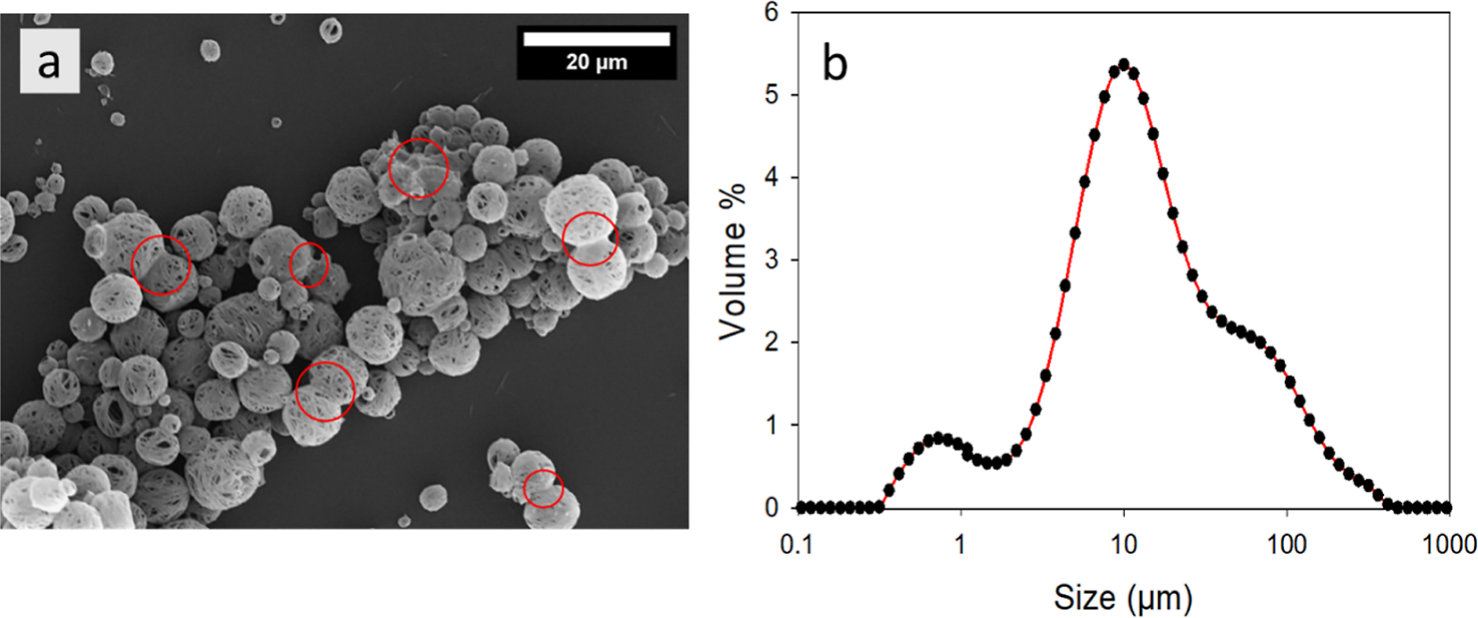
Microcapsules prepared using cyclohexyl isocyanate and 1,8-diaminooctane. (a) SEM image highlighting areas of partially fused capsules in red and (b) PSD of the same batch.

Although the optical and SEM images of microcapsules prepared using 1,8-diaminooctane revealed the size to be on the order of ∼2–10 μm (Figures 4a and 5b) for individual capsules, the particle size distribution (PSD, light scattering) of the microcapsules was slightly larger at 13 μm with a shoulder around 100 μm (SPAN = 6.2) (Figure 6b). Further investigation of the optical images of the microcapsule slurry revealed not only individual capsules but also larger aggregates of capsules (Figure 4a) which corroborated the PSD. To investigate this phenomenon, the stability of the emulsion (step 1 in Figure 2) was monitored over 24 h using light scattering. The PSD of the fresh emulsion (*t* = 0 h) overlapped with the PSD obtained after 24 h demonstrating that the emulsion itself was stable and the aggregation occurred during the shell formation, post the amine addition (step 2 in Figure 2). Upon closer observation of the SEM images, along with individual capsules, aggregates were also observed, wherein some of the capsules appeared to have partially fused, forming one large aggregate (red circles in Figure 6a).

**Figure 7:**
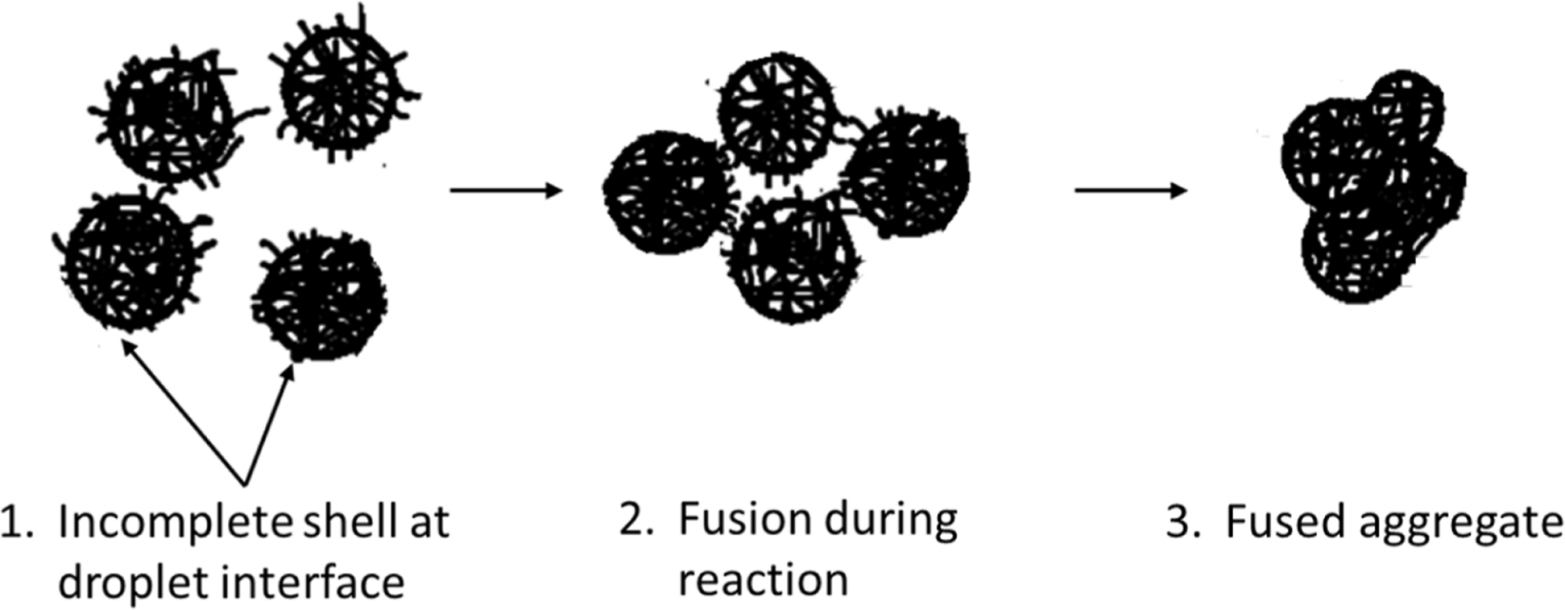
Proposed mechanism for aggregation during self-assembly.

Here, it is hypothesized that before the shell formation and reaction is complete (step 1, Figure 7), the incomplete shell at the interface of oil droplets pierces the stabilizing PVA layer of neighboring droplets destabilizing the emulsion (step 2, Figure 7). The incomplete shell present at the droplet interface prevents complete fusion. Subsequent self-assembly over partially fused droplets ultimately generates irregular aggregates rather than individual microcapsules (step 3, Figure 7). This phenomenon is commonly observed in food formulations like butter, where fat crystals pierce the interface of globules, which consequently forms aggregates.^50^ Similar phenomena have also been explained for oil-in-water emulsions in the presence of solids by McClements et al.^51^

### Using Laponite Nanodiscs

From the above, at least two limitations of these microcapsules were observed. First, they have a strong tendency to aggregate/fuse, and second, the shell is open and likely to be leaky. Therefore, a strategy was sought to both reduce the likelihood of aggregation and plug the open shell structure. Microcapsule aggregation can be reduced by the introduction of surface charge causing electrostatic repulsion between capsules, while leakiness can be addressed by either plugging the shell-surface voids or covering them with larger structures that cover the voids. To this end, Giermanska-Kahn et al. studied the use of nanoparticles to stabilize and control oil-in-water emulsions in the presence of fat crystals.^52^ They demonstrated that a layer of colloidal negatively charged silica particles adsorbed at the oil–water interface was sufficient to avoid partial coalescence caused by fat crystals growing in the droplets. Herein, Laponite nanodiscs were investigated not only as a moiety for introducing surface charge to inhibit capsule aggregation but also as a steric block to cover over the surface voids and enhance HS retention in the capsule.

**Figure 8:**
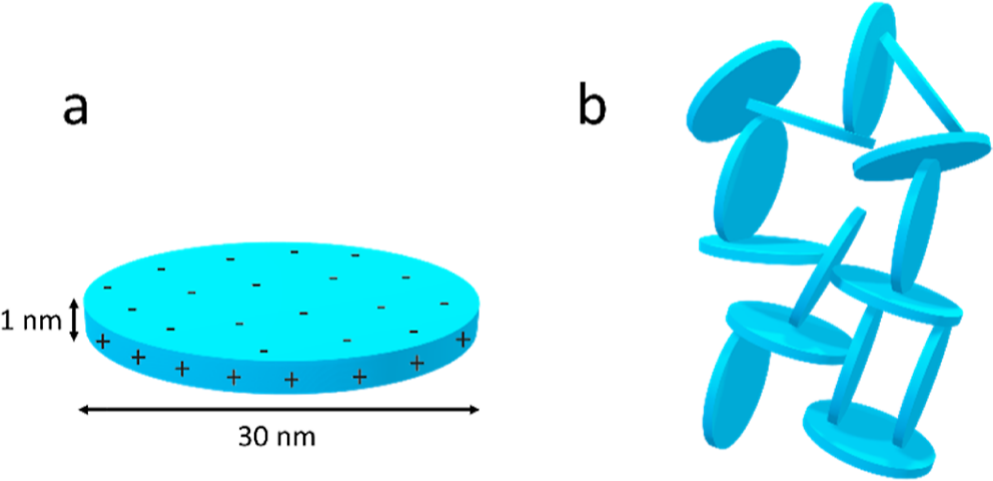
Laponite nanodiscs. (a) Illustration of an individual disc with dimensions and charge distribution across the disc surface (negative) and the rim (positive) and (b) their self-assembly in water leading to a “house of cards” structure.

Laponite nanoparticles are inorganic discs of roughly 30 nm diameter and 1 nm thickness (Figure 8a). Chemically, Laponite is a smectite clay that is made up of layers of octahedral sheets of magnesium oxide in between tetrahedral sheets of silica. Empirically, this can be represented as Na^+^_0.7_[(Si_8_Mg_5.5_Li_0.3_)O_20_(OH)_4_]^−0.7^. Lithium atoms substitute some of the magnesium resulting in a net negative charge commonly balanced by sodium ions.^53^ In water, the particles swell, releasing the sodium ions, making the disc surfaces negatively charged, while the OH^–^ ions on the rims become protonated, yielding slightly positively charged rims. This charge distribution causes the particles to align in a “rim to face” arrangement like a house of cards (Figure 8b), and as a result, Laponite nanodiscs tend to gel in water.

Studies highlighting the phase behavior of Laponite aqueous dispersions are described in the literature.^53,54^ The phase diagrams in these studies are depicted over a range of salt and Laponite concentrations. For Laponite nanodiscs, Ashby and Binks have demonstrated the formation of stable oil-in-water Pickering emulsions only when they are flocculated.^55^ The interparticle electrostatic repulsion inhibits flocculation when Laponite nanodiscs are dispersed in water. Salts like sodium chloride are added to partially block the surface charges and reduce electrostatic repulsion consequently forming flocs. These flocs concentrate at the oil–water interface during emulsification forming a barrier to coalescence.^56^

**Figure 9:**
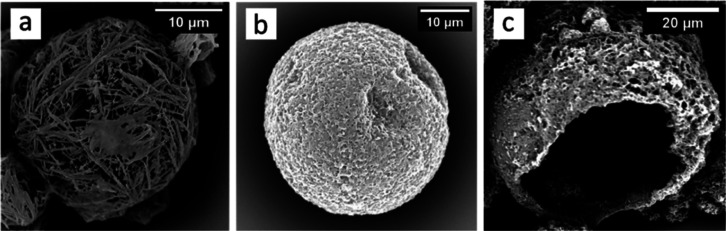
Morphology of microcapsule prepared using cyclohexyl isocyanate and 1,8-diaminooctane using (a) PVA and (b) Laponite nanodiscs as emulsion stabilizers and (c) cavity for microcapsules prepared using Laponite nanodiscs.

Keeping these factors in mind, we conducted preliminary encapsulation experiments, with cyclohexyl isocyanate and 1,8-diaminooctane as the precursors, using Laponite nanodiscs as Pickering emulsifiers, instead of PVA (step 1, Figure 2) in the presence of NaCl (0.01 M) in the continuous phase. Figure 9a shows the porous shell surface of microcapsules prepared using PVA as the emulsifier, which is in contrast to microcapsules prepared using Laponite nanodiscs as Pickering emulsifiers (Figure 9b,c) where the shell surface morphology is very different and continuous. Also, Figure 9c reveals a partially ruptured microcapsule with a core–shell structure and an oil-hosting cavity. To generate a sharper image of the shell cross-section in Figure 9c, the electron beam (15 kV) was focused on it for longer period than usual (∼90 s), which possibly damaged the shell surface making it rough and nonuniform (unlike the microcapsule in Figure 9b).

Dinkgreve et al. showed that emulsions stabilized by Laponite nanodiscs are not stable against shear and break down when subjected to flow.^57^ By adsorbing a dye onto the nanodiscs and observing via confocal microscopy, it was observed that they were heterogeneously dispersed in the continuous phase forming aggregated flocs which gel, and the emulsion droplets were dispersed in this gel. When the emulsion was subjected to stirring, the aggregated flocs broke down, destabilizing the emulsion. However, when a surfactant was used along with Laponite, a continuous network of nanodiscs was observed in the continuous phase with some nanodiscs present at the interface with the surfactant, providing stability during stirring.

**Figure 10:**
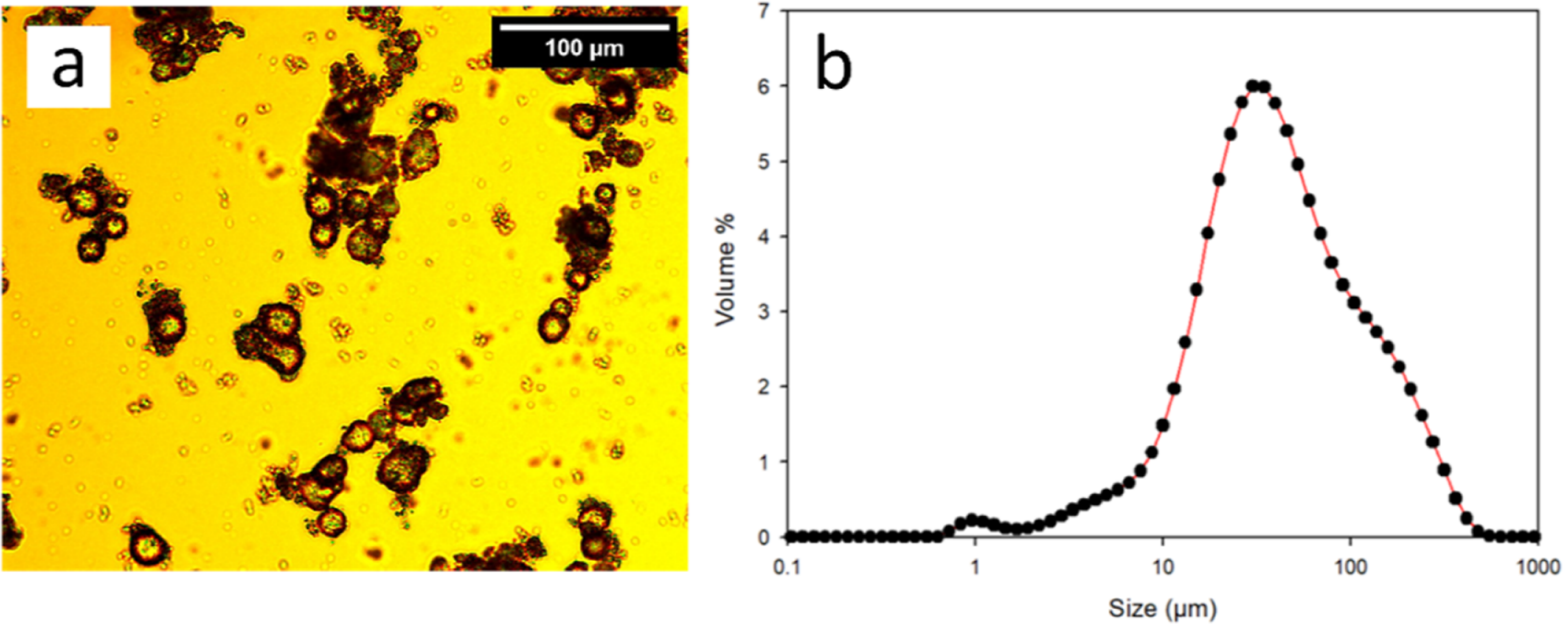
Microcapsules prepared using only Laponite nanodiscs for emulsification: (a) optical image and (b) PSD.

Our observations agreed with this study. When Laponite nanodiscs alone were used for stabilizing the emulsion, along with individual capsules, aggregates were also formed as shown in Figure 10. As the emulsion was stirred during amine addition, the Laponite gel stabilizing the emulsion must have broken down, destabilizing the emulsion.

**Figure 11:**
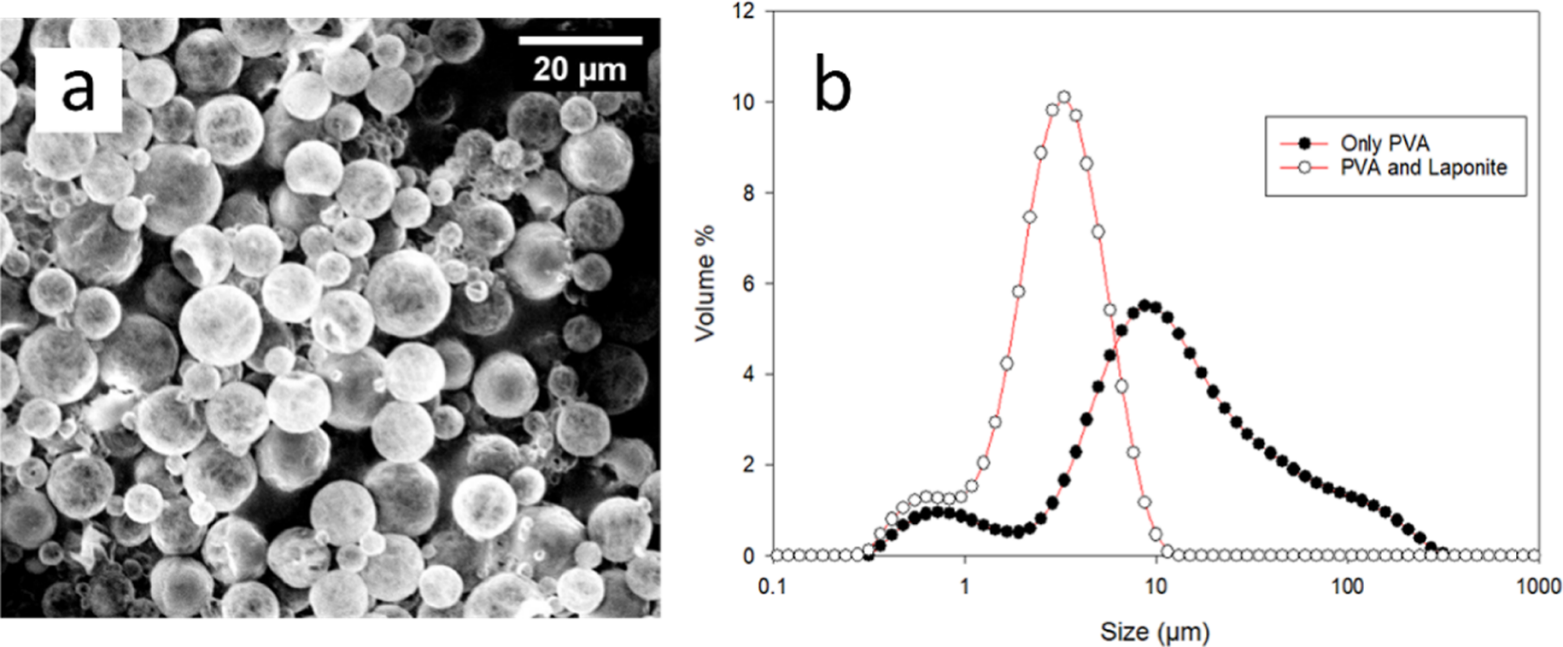
Microcapsules prepared using PVA and Laponite: (a) SEM image and (b) PSD.

To counter this effect, we used PVA along with Laponite nanodiscs to stabilize the emulsion. At an optimum concentration of 0.5% (w/w) PVA and 0.8% (w/w) Laponite, well-dispersed microcapsules (size distribution ∼1 to 10 μm and SPAN of 1.52) without aggregates were formed as shown in Figure 11. This size distribution is typical for emulsification systems formed using a rotor-stator type of homogenizer like the Silverson used here.^58^

**Figure 12:**
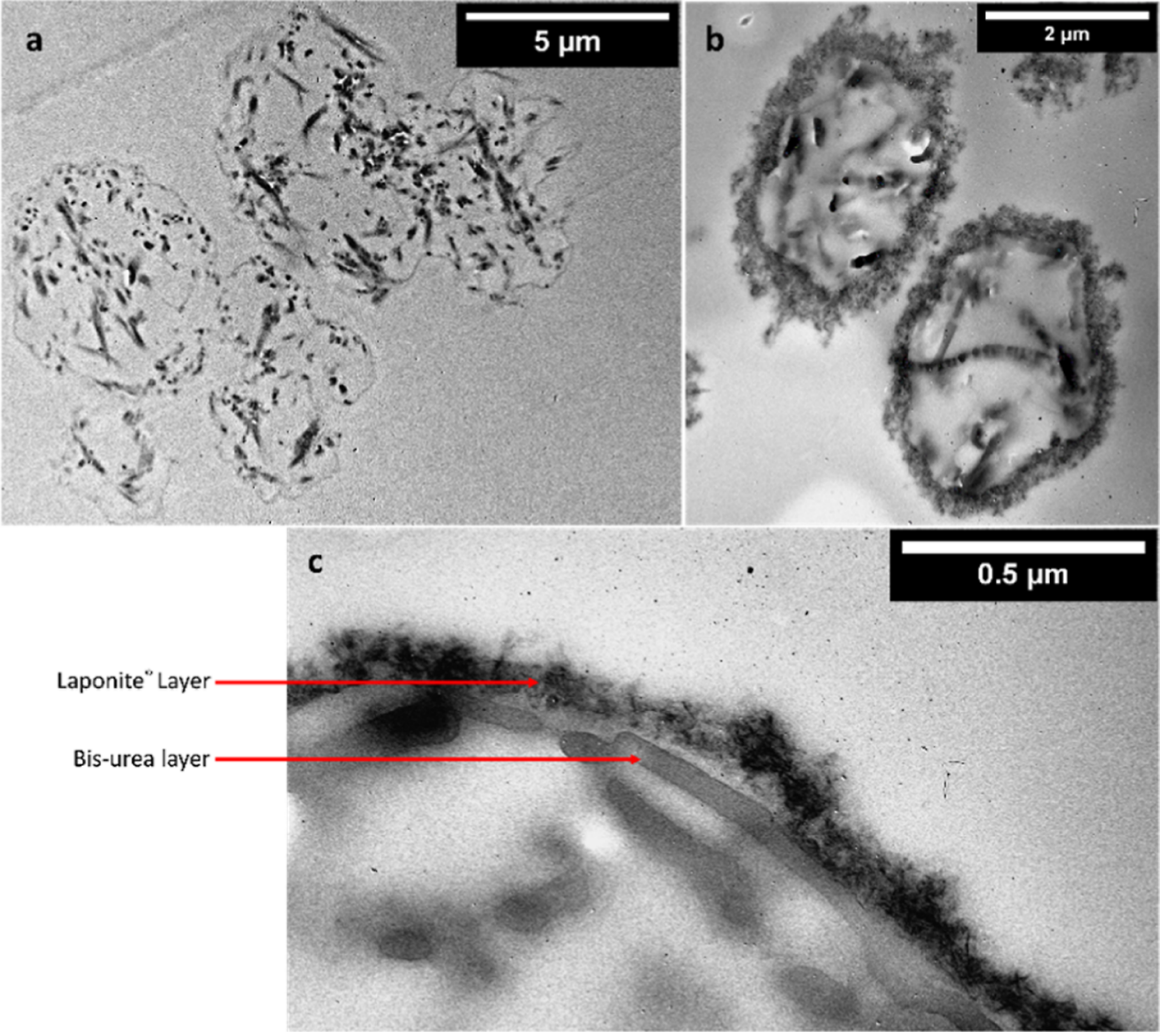
TEM images for microcapsules prepared using (a) PVA only, (b) PVA and Laponite, and (c) PVA and Laponite higher magnification for the shell.

To investigate the nature of the shell further, TEM images of the cross-section of the microcapsules were observed. As shown in Figure 12a, a clear shell boundary for isolated capsules is not visible, and the capsules appear to merge, forming aggregates, which corroborates our observation from previous SEM images (Figure 6a) for capsules prepared using PVA only. When the nanodiscs are introduced alongside the PVA, a layer of Laponite is formed around the capsule structure which prevents coalescence and aggregation during the self-assembly as shown in Figure 12b. With higher magnification, the fiber-like structure of the primary bis-urea shell structure is observed on the inside with an extra layer of Laponite nanodiscs on top (Figure 12c) which supports the change in morphology observed in the SEM images (Figures 9b and11a). Moreover, the distinct visibility of the primary bis-urea layer beneath the Laponite nanodiscs indicates that Laponite prevents aggregation of two or more capsules and may not affect the in situ self-assembly of the primary bis-urea shell. However, future work including independent investigations of both types of shell made using (i) PVA alone and (ii) PVA and Laponite would be required to confirm this, such as comparing their intrinsic structures using X-ray diffraction.^59^

### Payload and Encapsulation Efficiency

Table S1 shows the results of the intermediate steps of the calculation. The payload of capsules prepared using PVA only and capsules prepared using both PVA and Laponite nanodiscs was 70.0 ± 0.7 and 67.0 ± 0.2%, respectively (values after ± represent the standard error of the mean). Conventionally, payload is calculated based on the mass of core and shell components only. In our formula (eq 3), we use total mass of the sample since removing the protective colloid PVA completely is difficult during filtration. Therefore, if a weight percentage of all components in the formulation is considered on a dry basis, the payload is slightly underestimated by ∼2–3%. Both types of microcapsules prepared using (i) PVA alone and (ii) PVA and Laponite nanodiscs showed impressive EEs of 99.0 ± 0.2 and 99.0 ± 0.1%, respectively. Although the capsules prepared using PVA only appear to be porous (Figures 5b and 6a), they effectively encapsulate the oil in aqueous conditions and hold it during filtration. The solubility of HS in water is very low (∼10^–6^ g mL^–1^),^60^ indicating that the driving force for its diffusion across the capsule shell into the aqueous medium is small. Also, it has a high boiling point (290 °C), which further minimizes losses by evaporation.

### Release Profile

**Figure 13:**
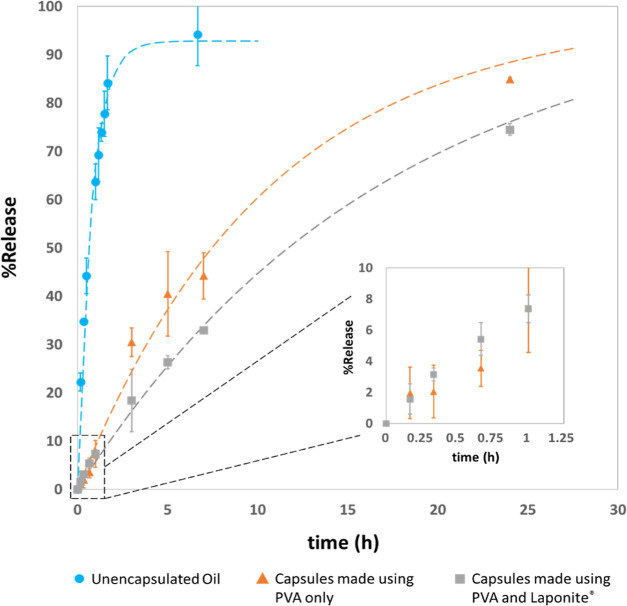
Release profiles of HS from both types of microcapsules in 36% (v/v) aqueous 1-propanol, the error bars represent the standard error of the mean. Inset shows a magnified release profile over the first hour. The dotted lines display the release profile generated using the model *y* = *a* (1–*e*^(−*t*/τ)^).

The accelerated release rate of HS from the capsules was recorded in 36% (v/v) aqueous 1-propanol, in which the solubility of HS is ∼5.2 × 10^–3^ g mL^–1^.^60^ This is almost 3 orders of magnitude higher than the solubility of HS in pure water. Therefore, the driving force for the release of encapsulated HS into this 1-propanol solution was higher as compared to pure water, ensuring that the release experiment was completed within a reasonable time frame. The capsules (and unencapsulated HS in the control experiment) were contained inside a dialysis tube during the experiment. This containment was crucial to avoid the withdrawal of the capsules along with the aliquots. Figure 13 shows the release profiles. As seen clearly, about 90% of all HS is released within 6 h when it is unencapsulated, which indicates that the mass transfer resistance from the dialysis tube is small but not negligible. The capsules prepared using only PVA offer a significant resistance to release with only about 40% of oil released in 6 h. The release rate of HS from the capsules prepared using PVA and Laponite nanodiscs is only slightly slower than the capsules prepared using only PVA. However, when we compare the size of the capsules in both these batches, the capsules prepared using only PVA are much larger and aggregated (mean Sauter diameter *d* [3,2] = 5.2 μm), whereas the capsules prepared using PVA and Laponite are smaller and well dispersed (*d* [3,2] = 2.3 μm). Effectively, the total interfacial area (inversely proportional to *d* [3,2]) available for mass transfer is higher for the latter. Thus, the resistance to release offered by the capsule shell prepared using PVA and Laponite would be much higher than that offered by the capsules prepared by using only PVA.

### Mathematical Modeling of the Release Profiles

To further understand the accelerated release rates and quantify the impact of encapsulation on rate reduction, an exponential equation is used for modeling the release curves in terms of the characteristic time τ for release
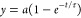
4where *y* is the cumulative release of HS (weight fraction) in time *t* and *a* is a dimensionless constant scaling the released oil. Using the built-in solver function in Microsoft Excel and least-squares analysis, this equation was used to fit the release data. Table 1 shows the values obtained for *a* and τ. As expected, the characteristic time for both types of capsules (3.8 × 10^4^ and 6.1 × 10^4^ s) is longer by more than an order of magnitude than that for the unencapsulated oil (2.8 × 10^3^ s) which signifies the increase in mass transfer resistance offered by the microcapsule shell. Furthermore, the extra layer of laponite nanodiscs offers additional resistance to leakage and consequently, the characteristic time is ∼2.3 × 10^4^ s longer than microcapsules prepared using PVA alone.

**Table 1:** Constants Obtained after Fitting *y* = *a* (1–*e*^–*t*/τ^) to the Release Profiles

	*a*	τ (s)	*R*^2^
unencapsulated oil	0.92	2.8 × 10^3^	0.99
capsules made using PVA only	0.98	3.8 × 10^4^	0.99
capsules made using PVA and Laponite	1	6.1 × 10^4^	0.99

Since *y* in eq 4 represents the weight fraction, theoretically, at *t* = ∞, when all oil is released, *y* = *a* = 1. Experimentally, the concentration gradient between inside the capsules (or inside the dialysis tube for unencapsulated oil) and the outer bulk is the major driving force for release. As oil is released in the bulk, this gradient reduces until finally an equilibrium is attained with a uniform concentration throughout, and some amount of oil (∼2–5%) can remain inside the dialysis tube. This, along with the margin of error in the experiment, might explain the value of *a* not being equal to 1 in the first two cases (as seen in Table 1). Nevertheless, they are close to 1 (0.92 and 0.98), which is reasonable.

### Mechanical Strength of the Capsules

**Figure 14:**
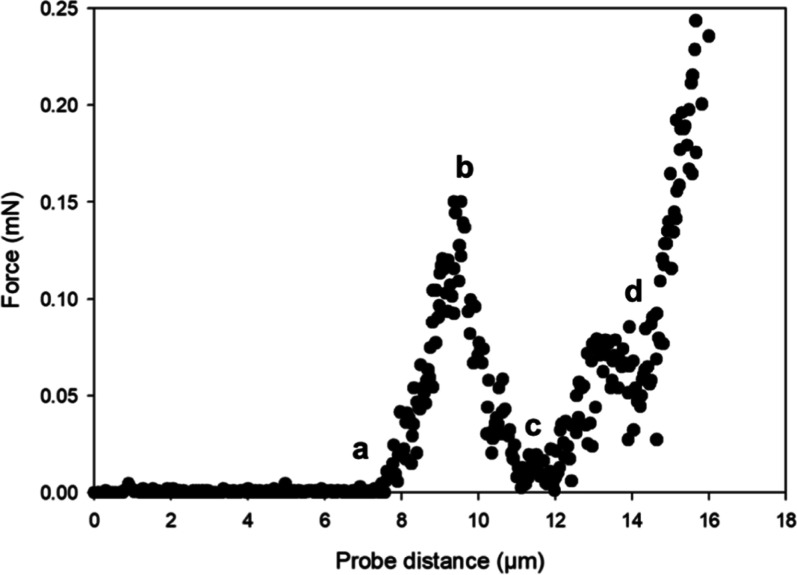
Typical curve of force vs distance traveled by probe during compression of a single microcapsule (diameter = 7 μm), obtained from the micromanipulation rig for microcapsules prepared using PVA and laponite.

Figure 14 shows a typical graph of force acting on a capsule vs the distance moved by the probe generated for compressing one capsule using the micromanipulation technique. The curve from 0 to “*a*” corresponds to the probe moving in air. At point “*a*”, the probe touches the capsule, and as it moves further, the capsule gets compressed with an increase in force until, eventually, it ruptures at point “*b*”. As a result, the force drops to point “*c*”. From point “*c*” to “*d*”, the probe continues to compress the broken shell/debris of the capsule until finally, at point “*d*”, it starts to push onto the glass substrate, leading to a rapid increase in force. Using this curve, the rupture force (*F*_R_) was determined (the force at point *b*). Also, the displacement at rupture (δ_R_) is the distance traveled by the probe once it touches the capsule until rupture. Using these values, for a capsule with diameter *d*, the following mechanical strength parameters can be determined^49^
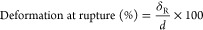
5
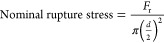
6
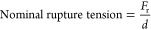
7

**Figure 15:**
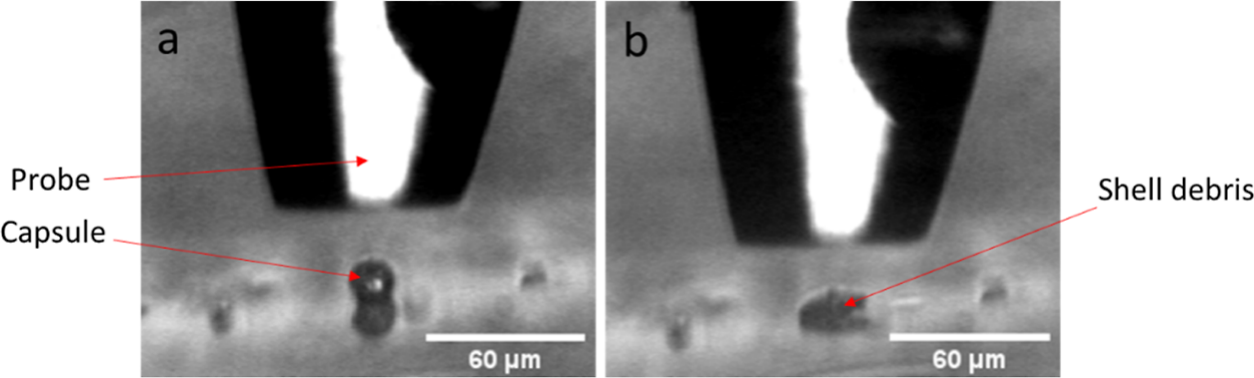
Image obtained from the micromanipulation rig (a) before and (b) after rupturing the capsule.

Figure 15 shows the image obtained from the micromanipulation rig, and Table 2 summarizes the mechanical strength parameters determined for capsules made using (i) PVA alone and (ii) PVA and Laponite nanodiscs. A clear improvement in the rupture force, rupture tension, and nominal rupture stress is observed upon using the Laponite nanodiscs which may be attributed to the extra layer of Laponite formed around the capsules.

**Table 2:** Summary of the Mechanical Strength Parameters of Microcapsules^a^

	with PVA only	with PVA and Laponite
mean diameter, *d* (μm)	9.0 ± 0.7	6.8 ± 0.6
rupture force, *F*_r_ (mN)	0.07 ± 0.02	0.15 ± 0.02
nominal rupture tension (μN/μm)	7 ± 1	23 ± 2
displacement at rupture,δ_R_ (μm)	1.6 ± 0.3	1.0 ± 0.2
deformation at rupture (%)	18 ± 2	15 ± 4
nominal rupture stress (MPa)	1.0 ± 0.1	4.6 ± 1.1
number of capsules measured	30	30

a The figure after ± represents the standard error of the mean.

Guinebretiere et al. patented UF and MF microcapsules that were used in commercial formulations (e.g., detergents, fabric conditioners, dishwashing liquid).^61^ These microcapsules were tested by using the same methodology. A range of volume-weighted nominal rupture stresses from 0.1 to 16 MPa were outlined wherein capsules can be used for specific industrial products based on their mechanical strengths. The mean diameter of these commercial microcapsules varied from 16 to 31 μm which differs from the capsules prepared in this work, making a straightforward comparison challenging. Thus, using eqs 6 and 7, the rupture tension for these UF and MF microcapsules was calculated to normalize the effect of size, which was ∼7 to 111 μN/μm. Previously, MF microcapsules prepared by Long et al. had a rupture tension of 72 ± 6 μN/μm.^49^ The two types of bis-urea microcapsules prepared here (with rupture tensions equal to 7 ± 1 and 23 ± 2 μN/μm) have mechanical strength parameters comparable to those of some of the commercial polymeric microcapsules, although there is a scope for further improvement. However, it is important to note that the bis-urea shell prepared in this work is inherently formed using noncovalent bonds, whereas the synthetic polymeric shells used in commercial microcapsules are highly cross-linked covalently bonded networks. Consequently, the latter are more difficult to break under tension and more difficult to degrade, leading to greater mechanical strength. It should be highlighted that the bis-urea microcapsules were stable when exposed to dry (during micromanipulation) and aqueous conditions and can work well for applications (e.g., agriculture, laundry, and homecare) where similar conditions prevail. However, if the environmental conditions are significantly different, due to the noncovalent nature of the shell, these microcapsules will have to be tested in conditions relevant to the final application.

## Conclusions

A novel method to create microplastic-free microcapsules using the self-assembly of small organic bis-urea molecules was demonstrated successfully in this work. By optimization of the precursors used in the reaction, the self-assembly of the molecules was tuned to generate a shell at the oil–water interface. Laponite nanodiscs were incorporated into the formulation to improve the stability of the emulsion during shell formation. A uniform layer of Laponite was formed around the primary capsule shell, plugging the open pores and restricting particle aggregation to form well-dispersed microcapsules. Both types of microcapsules, prepared using (i) PVA alone and (ii) PVA and Laponite, offered excellent EE (∼99%) when encapsulating HS. Furthermore, more than an order of magnitude increase in characteristic release time as compared to unencapsulated HS was observed in both types of microcapsules. Specifically, the extra layer of Laponite successfully improved the barrier properties of the capsules and led to a further increase in characteristic release time by 2.3 × 10^4^ s. Considering the noncovalent nature of the shell, the capsules showed reasonable mechanical strength (nominal rupture tension ∼7–23 μN/μm). Although there remains a scope for further improvement, these mechanical strengths are comparable to those of some of the conventional polymeric microcapsules used in commercial products. All in all, this technique can be a viable alternative to make microplastic-free microcapsules industrially under benign conditions. Furthermore, it opens possibilities to test a plethora of small organic molecules for encapsulation using an organic solvent-free, one-pot process. In principle, if the functionality of precursors is restricted, all of the chemistries conventionally used for making microcapsules using interfacial polymerization can be tested using this new method.

## Supporting Information Available

Tabulated results for payload and EE and details of experiments conducted using 1,10-diaminodecane and 1,12-diaminododecane (PDF)
