# Supplementary material for: Microplastic-Free Microcapsules Using Supramolecular Self-Assembly of Bis-Urea Molecules at an Emulsion Interface
Source: Langmuir. 2024 Jul 11;40(29):14798–810. doi: 10.1021/acs.langmuir.4c00541 (PMC11270993; doi:10.1021/acs.langmuir.4c00541)
Supplement: Supplementary file 1 — la4c00541_si_001.pdf [file la4c00541_si_001.pdf]

Microplastic-free microcapsules using supramolecular self-assembly of bis-urea molecules at an emulsion interface

Siddhant Pravin Bhutkar†, Pierre-Eric Millard§, Jon A. Preece‡ and Zhibing Zhang*, †

†School of Chemical Engineering, University of Birmingham, Birmingham B15 2TT, UK
‡School of Chemistry, University of Birmingham, Birmingham B15 2TT, UK
§BASF SE, 67056 Ludwigshafen am Rhein, Germany
*Corresponding author, email: z.zhang@bham.ac.uk

Section S1: Payload and Encapsulation Efficiency

Table S1 shows the summary of the results obtained during all the calculations done for obtaining the payload and encapsulation efficiency of hexyl salicylate (HS) for both types of capsules (performed in duplicates).

Section S2: Experiments using 1,10 – diaminodecane and 1,12 – diaminododecane

Capsule synthesis experiments were performed using 1,10 – diaminodecane and cyclohexyl isocyanate as the reagents (isocyanate:amine molar ratio = 2:1) . Physically, 1,10 – diaminodecane is a white powder sparingly soluble in water. 1.4 g of diaminodecane was added to the stable oil-in-water emulsion (*Step 1*, **Figure 2** in the manuscript). **Figure S1a** shows the optical image of the reaction mixture observed after 2 h of the diamine addition. The image shows large lumped masses with some smaller droplets which could be incompletely formed microcapsules. Furthermore, the solid powder was still seen to be floating in the emulsion. The reaction mixture was stirred overnight (18 h), and large lumps were observed (**Figure S1b**).

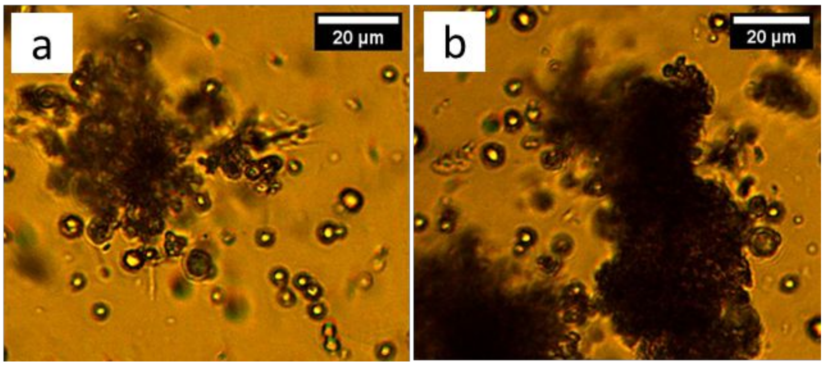

Figure S1: Optical images of reaction mixture after (a) 2 h and (b) 20 h, when 1,10-diaminodecane – cyclohexyl isocyante were used as reagents.

Table S1: Summary of results obtained during payload and encapsulation efficiency experiments.

		PVA only		PVA and Laponite®	
	Duplicates	A	B	A	B
Encapsulation Efficiency	Unencapsulated HS extracted into dibutyl adipate ($\mathbf{W_{1}}$, mg)	1.6	2.3	1.9	1.6
	Total amount of HS present in 2 g microcapsule slurry ($\mathbf{W_{total}}$, mg)	193.2	196.8	179.4	174.7
	Mean $\mathbf{W_{total}}$, mg	195		177	
	Encapsulation Efficiency **EE %** = $((W_{total}-W_{1})/W_{total})*100$	99.2	98.8	98.9	99.1
	Mean **EE%**	99		99	
	Standard Error %	0.2		0.1	
Payload	Mass of dry capsules used ($\mathbf{mass_{sample}}$, mg)	106	103	105.5	101.5
	HS extracted into isopropanol ($\mathbf{mass_{core}}$, mg)	73.8	73.3	71.8	68.7
	**Payload**, % $(mass_{core}/mass_{sample})*100$	69.7	71.1	68.1	67.6
	Mean **Payload %**	70.4		67.9	
	Standard error %	0.7		0.2	

Similar observations were made when 1,12-diaminododecane and cyclohexyl isocyanate were used as reagents. 1.6 g of 1,12 -diaminododecane powder was added to the oil-in-water emulsion (*Step 1*, **Figure 2** in the manuscript). **Figure S2** shows the optical images of the reaction mixture after 2 h and 20 h of stirring.

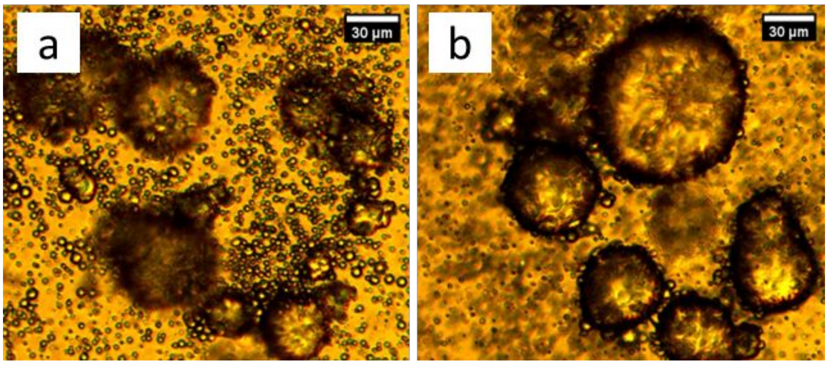

Figure S2: Optical images of reaction mixture after (a) 2 h and (b) 20 h, when 1,12-diaminododecane – cyclohexyl isocyante were used as reagents.

In both these experiments, three steps were presumably occurring: (1) dissolution of the diamine into water until saturation (2) reaction of the dissolved amine with the isocyanate in the dispersed phase and (3) further dissolution of the solid amine. Size of the diamine powders itself was large enough to hamper the stability of the original emulsion and the presence of these solids could also affect the supramolecular self-assembly, which may contribute to the formation of large lumps. Moreover, it was difficult to judge whether these large particles were unreacted diamine particles or aggregated bis-ureas.

An alternative method to judge the effect of alkyl chain lengths on the supramolecular self-assembly of bis-urea molecules would involve a multi-step approach: (1) Separately prepare the bis-urea molecule using an organic solvent (2) separate the pure bis-urea powder (3) dissolve the bis-urea powder along with HS in a volatile organic solvent (*e.g.* DCM) (4) Emulsify this organic solution in water and let the volatile solvent phase separate and evaporate. Here, the interfacial behaviour of the bis-urea molecule can be effectively studied and the influence of alkyl chain length on the interfacial assembly can be determined. However, the main aim of this work was to prepare microplastic-free microcapsules using a benign, one-pot process without using any organic solvent. Therefore, these two experiments conducted using 1,10 – diaminodecane and 1,12-diaminododecane were not studied further.
